# Supplementary material for: Where to invest in neonatal survival programs in Nepal? A modelling study using Lives Saved Tool through scaling key interventions
Source: PLoS One. 2025 Dec 4;20(12):e0337331. doi: 10.1371/journal.pone.0337331 (PMC12677573; doi:10.1371/journal.pone.0337331)
Supplement: S1 File — (DOCX) [file pone.0337331.s001.docx]

**Supplementary Information**

# **Where to Invest in Neonatal Survival Programs in Nepal? A Modelling Study Using Lives Saved Tool Through Scaling Key Interventions**

**Authors and affiliation**

Geha Nath Khanal^1,2*^, Nisha Giri^3^, Deepak Jha^4^, Dipak Raj Chaulagain^5^

^1^ Nepal Public Health Association, Lalitpur, Nepal

^2^ School of Nursing, Midwifery, Allied and Public Health, Canterbury Christ Church University Canterbury, United Kingdom

^3^ Nursing Association of Nepal, Kathmandu, Nepal

^4^ Ministry of Health and Population, Department of Health Services, Family Welfare Division, Kathmandu, Nepal

^5^ Institute for Implementation Science and Health, Kathmandu, Nepal

*Corresponding author

Nepal Public Health Association, Lalitpur, Nepal

Email: [khanalg@outlook.com](mailto:khanalg@outlook.com)

**Supplementary Information**

**S1 Table:** Summary of indicators, their definitions, baseline and targets, and data sources

*S1 Table: Summary of indicators, their definitions, baseline and targets, and data sources*

| **SN** | **Indicator** | **Definition** | **Baseline** | **Target*** | **Data source for baseline** | **Proxy indicator** |
| --- | --- | --- | --- | --- | --- | --- |
| 1 | Folic acid supplementation/fortification | Percent of women 15-49 that are taking folic acid supplements (5.0 mg folic acid per day) or have appropriate food fortification around the time of pregnancy. | 4.01 | 50 | Supplement to (Nove et al., 2021) | 5% of ANC 4+ receive folic acid supplementation |
| 2 | Safe abortion services | Among women who get an abortion, the percent who get a safe abortion (defined as via D&C, vacuum aspiration, or medical abortion). | 48 | 60 | (Ghimire et al., 2024) |  |
| 3 | Post-abortion case management | Percent of women who have had an abortion who get the appropriate postabortion case management at a Basic Emergency Obstetric Care (BEmOC) level. | 59.55 | 60 | Supplement to (Nove et al., 2021) | if facility delivery is >50%, 0.75 × facility delivery; if facility delivery is 30–50%, 0.50 × facility delivery; if facility delivery is <30%, 0.10 × facility delivery |
| 4 | Antenatal care | Percent of women who attend four or more antenatal care visits during their pregnancy (ANC 4+). | 80.2 | 95 | (Ministry of Health and Population et al., 2023), Table 9.2 |  |
| 5 | TT - tetanus toxoid vaccination | Percent of women who received two doses of tetanus toxoid during this pregnancy | 58.2 | 95 | (Ministry of Health and Population et al., 2023), Table 9.8 |  |
| 6 | Syphilis detection and treatment | Percent of pregnant women tested for syphilis and given treatment if needed. | 3.4% | 95 | (Kanyangarara & Chou, 2017), (Table 2) | -1.22 + 3.36 X (blood sample) + -5.23(Early ANC) |
| 7 | Iron Folate supplementation | Percent of pregnant women taking an iron supplement daily, for at least 90 days. | 86.50 | 95 | (Kanyangarara & Chou, 2017), (Table 2) | Percentage of pregnant women receiving ≥90 days of iron folate |
| 8 | Hypertensive disorders case management | Percent of women receiving detection and appropriate management of moderate to severe hypertension during pregnancy. | 63.8% | 80 | (Kanyangarara & Chou, 2017), (Table 2) | Proxy formula: -1.62 + 2.5 (urine sample) |
| 9 | Diabetes case management | Percent of pregnant women screened for diabetes and managed appropriately, if needed. | 26.4% | 80 | (Kanyangarara & Chou, 2017), (Table 2) | Proxy formula: -3.21 + 2.61 (blood sample) |
| 10 | MgSO4- management of pre-eclampsia | Percent of pregnant women with pre-eclampsia who are treated with intravenous magnesium sulfate (4-6g). | 43.3% | 80 | (Kanyangarara & Chou, 2017), (Table 2) | Proxy formula: -6.44 + 4.91 (blood sample) + 2.56 (ANC4+ |
| 11 | Skilled birth attendance (SBA) | Percent of children born with a skilled attendant present, including doctors, nurses, or midwives, in a facility or at home. | 80.1 | 95 |  |  |
| 12 | Institutional delivery | Percent of children born in a health facility. | 79.4 | 95 |  |  |
|  | *Essential Care* | *% of infants delivered in a birthing centre* | 29.67 |  | HMIS Report (2080/81) |  |
|  | *BEmONC* | *% of infants delivered in a BEmONC level health facility* | 7.07 |  | HMIS Report (2080/81) |  |
|  | *CEmONC* | *% of infants delivered in a CEmONC level health facility* | 63.26 |  | HMIS Report (2080/81) |  |
| 13 | Clean birth practices | Percent of deliveries where clean birth practices are performed, including handwashing by the attendant, cleaning the maternal perineum, using a clean birth surface, clean cutting and tying of the cord, and hygienic cord and skin care immediately after delivery. | 79.4 | 95 | (LiST Team, 2024) | 100% of institutional deliveries are using clean birth practices (LiST Team, 2024) |
| 14 | Immediate assessment and stimulation | Percent of deliveries where rubbing and drying of the neonate immediately after delivery is performed. | 79.4 | 95 |  | 100% of institutional deliveries are using immediate assessment and stimulation (LiST Team, 2024) |
| 15 | Labor and delivery management | Percent of women receiving labor and delivery management from a skilled birth attendant. | 79.4 | 95 |  | 100% of institutional deliveries have access to the appropriate facilities for the given level of care (LiST Team, 2024) |
| 16 | Antibiotics for preterm premature rupture of membranes (PPRoM) | Percent of pregnant women with premature rupture of the membranes (pPRoM) who are not in labor and are given oral erythromycin (250mg, 4 times daily for 7 days) to prevent infection. | 70.33 | 95 |  | The default assumption is that 100% of BEmOC and 100% of CEmOC deliveries have access to antibiotics for preterm prelabor rupture of membranes, if needed (LiST Team, 2024) |
| 17 | MgSO4 - management of eclampsia | Percent of pregnant women receiving magnesium sulfate for eclampsia during delivery. | 70.33 | 95 |  | The default assumption is that 100% of BEmOC and 100% of CEmOC deliveries have access to MgSO4 for eclampsia, if needed (LiST Team, 2024) |
| 18 | AMTSL - Active Management of the Third Stage of Labor | Percent of women with their third stage of labor managed actively. Active management of the third stage of labor (AMTSL) is defined as controlled cord traction, uterine massage, and appropriate oxytocics. | 70.33 | 95 |  | The default assumption is that 100% of BEmOC and 100% of CEmOC deliveries have appropriate active management during and after delivery (LiST Team, 2024) |
| 19 | Induction of labor for pregnancies lasting 41+ weeks | Percent of women who are 41 or more weeks pregnant who are managed with induction of labor as appropriate. | 63.26 | 95 |  | 100% of CEmOC deliveries have access to induction of labor for post-term pregnancies, if needed (LiST Team, 2024) |
| 20 | Case management of premature babies | This refers to the sum of the three levels of management of prematurely born infants in the neonatal period: thermal care, Kangaroo mother care, and full supportive care for prematurity. *See below for details on each intervention.* | 29.9 | 65* | (Department of Health Services, 2025), Table 3.5 |  |
| 21 | Case management of severe neonatal infection | Sum of the three levels of case management for severe infection in the neonatal period: oral antibiotics, injectable antibiotics, and full supportive care. See below for details on each intervention. | 71 | 100 |  |  |
| 22 | Neonatal resuscitation for asphyxia | Percent of deliveries that require opening the airway and ventilating newborn babies who do not spontaneously cry/breathe after birth | 29.6 | 85 | (Tuladhar et al., 2024) | Calculated from service availability analysis from NHFS 2021 |
| 23 | Exclusive breastfeeding | Percent of children receiving only breastmilk for food (plus medication, vaccines, and vitamins). | 56.4 | 90 | (Ministry of Health and Population et al., 2023), Table 11.8 |  |
| 24 | Clean Postnatal Practcies | Percent of neonates where the mother washes her hands frequently, the child lives in a clean environment, and no harmful practices are performed. | 69.7 | 95 | (Ministry of Health and Population et al., 2023), Table 9.20 | % of neonates receiving a preventive postnatal visit within 48 hours of delivery |
| 25 | Chlorhexidine | Percent of neonates with chlorhexidine applied after birth to the cord. | 51.2 | 95 | (Ministry of Health and Population et al., 2023), Table 9.27 |  |
| 26 | Complementary feeding - education only | Percent of mothers intensively counseled on the importance of continued breastfeeding beyond six months and appropriate complementary feeding practices. As a proxy, the percent of 6-23 month old children receiving minimum dietary diversity (4+ food groups) is used. | 48.7 | 95 | (Ministry of Health and Population et al., 2023), Table 9.27 | As a proxy, the percent of 6-23 month old children receiving minimum dietary diversity (4+ food groups) is used (LiST Team, 2024) |
| 27 | Complementary feeding - education and supplementation | Percent of mothers intensively counseled on the importance of continued breastfeeding beyond six months and appropriate complementary feeding practices, and given appropriate dietary supplementation. As a proxy, the percent of 6-23 month old children receiving minimum dietary diversity (4+ food groups) is used. | 48.7 | 95 | (Ministry of Health and Population et al., 2023), Table 9.27 | As a proxy, the percent of 6-23 month old children receiving minimum dietary diversity (4+ food groups) is used (LiST Team, 2024) |
| 28 | Improved water source | Percent of households with access to an improved water source within a 30 minute walk. | 94.9 | 95 | (National Planning Commission, 2020) Table WS 1.2 |  |
| 29 | Water connection in the home | Percent of households with a household connection, including water piped into the home or yard. | 36 | 50 | (National Planning Commission, 2020) Table WS 1.1 |  |
| 30 | Improved sanitation | Percent of households using an improved sanitation facility (defined as flush or pour flush to piped sewer system, septic tank, or pit latrine; ventilated improved pit (VIP) latrine; pit latrine with slab; or composting toilet). | 93.8 | 95 | (National Planning Commission, 2020) Table WS 3.1 |  |
| 31 | Hand washing with soap | Percent of mothers using appropriate hand washing practices, including washing hands with soap, ash, or other materials and using adequate water, after handling feces and before preparing food. | 80.7 | 95 | (National Planning Commission, 2020), Table WS 2.1 |  |
| 32 | Hygienic disposal of children's stools | Percent of children's stools that are disposed of safely and contained. Stools are considered to be contained if: 1) the child always uses a toilet/latrine, 2) the feces are thrown in the toilet/latrine, or 3) the feces are buried in the yard. | 70.3 | 95 | (National Planning Commission, 2020) Table WS 3.5 |  |
| 33 | ORS - oral rehydration solution | Percent of children 0-59 months with suspected diarrhea treated with oral rehydration solution (ORS), including sachets or pre-mixed solutions. | 38 | 90 | (Ministry of Health and Population et al., 2023), Table 10.13 |  |
| 34 | Zinc - treatment of diarrhea | Percent of children 0-59 months with suspected diarrhea treated with 20mg of zinc daily. | 17.9 | 90 | (Ministry of Health and Population et al., 2023), Table 10.13 |  |
| 35 | Oral antibiotics for pneumonia | Percent of children with suspected pneumonia (symptoms of acute respiratory infection) for whom advice or treatment was sought from a health facility or provider. | 75 | 90 | (Ministry of Health and Population et al., 2023), Table 10.7 (page 250) |  |

Note: * The target values are extracted from (Ministry of Health, 2016)

**References**

Department of Health Services. (2025). *Annual Health Report 2080/81 (2023/24)*. Ministry of Health and Population. https://hmis.gov.np/media/40/Annual-Health-Report-208081_compressed.pdf

Ghimire, J., Lal, B. K., Karki, S., Mehata, S., Dotel, B. R., Joshi, N., Shrestha Pradhan, G., Gautam, M., Shrestha Dangol, D., Phuyal, P. P., Lamichhane, K., Bhattarai, N., & Lynam, A. (2024). An Estimate of Abortion Incidence and Unintended Pregnancies. *Journal of Nepal Health Research Council*, *22*(1), 50–57. https://doi.org/10.33314/jnhrc.v22i01.4945

Kanyangarara, M., & Chou, V. B. (2017). Linking household surveys and health facility assessments to estimate intervention coverage for the Lives Saved Tool (LiST). *BMC Public Health*, *17*(S4), 780. https://doi.org/10.1186/s12889-017-4743-4

LiST Team. (2024). *Spectrum Manual Spectrum System of Policy Models*. https://avenirhealth.org/Download/Spectrum/Manuals/SpectrumManualE.pdf

Ministry of Health. (2016). *Nepal’s Every Newborn Action Plan*. https://www.healthynewbornnetwork.org/hnn-content/uploads/NENAP-final-low-resolution.pdf

Ministry of Health and Population, New Era, & ICF. (2023). *Nepal Demographic and Health Survey 2022*. https://www.dhsprogram.com/pubs/pdf/FR379/FR379.pdf

National Planning Commission. (2020). *Nepal Multiple Indicator Cluster Survey 2019*. https://www.unicef.org/nepal/media/11081/file/Nepal%20MICS%202019%20Final%20Report.pdf

Nove, A., Friberg, I. K., De Bernis, L., McConville, F., Moran, A. C., Najjemba, M., Ten Hoope-Bender, P., Tracy, S., & Homer, C. S. E. (2021). Potential impact of midwives in preventing and reducing maternal and neonatal mortality and stillbirths: A Lives Saved Tool modelling study. *The Lancet Global Health*, *9*(1), e24–e32. https://doi.org/10.1016/S2214-109X(20)30397-1

Tuladhar, S., Paudel, D., Rehfuess, E., Siebeck, M., Oberhauser, C., & Delius, M. (2024). Changes in health facility readiness for obstetric and neonatal care services in Nepal: An analysis of cross-sectional health facility survey data in 2015 and 2021. *BMC Pregnancy and Childbirth*, *24*(1), 79. https://doi.org/10.1186/s12884-023-06138-8
