# Supplementary material for: Where to invest in neonatal survival programs in Nepal? A modelling study using Lives Saved Tool through scaling key interventions
Source: PLoS One. 2025 Dec 4;20(12):e0337331. doi: 10.1371/journal.pone.0337331 (PMC12677573; doi:10.1371/journal.pone.0337331)
Supplement: S2 File — (DOCX) [file pone.0337331.s002.docx]

**Supplementary File**

# **Where to Invest in Neonatal Survival Programs in Nepal? A Modelling Study Using Lives Saved Tool Through Scaling Key Interventions**

**Authors and affiliation**

Geha Nath Khanal^1,2*^, Nisha Giri^3^, Deepak Jha^4^, Dipak Raj Chaulagain^5^

^1^ Nepal Public Health Association, Lalitpur, Nepal

^2^ School of Nursing, Midwifery, Allied and Public Health, Canterbury Christ Church University Canterbury, United Kingdom

^3^ Nursing Association of Nepal, Kathmandu, Nepal

^4^ Ministry of Health and Population, Department of Health Services, Family Welfare Division, Kathmandu, Nepal

^5^ Institute for Implementation Science and Health, Kathmandu, Nepal

*Corresponding author

Nepal Public Health Association, Lalitpur, Nepal

Email: [khanalg@outlook.com](mailto:khanalg@outlook.com)

**Supplementary Tables for detail results by year, 2025 to 2035**

**S1 Table:** Additional neonatal lives saved by cause between 2025 and 2035

**S2 Table:** Neonatal mortality rate by year between 2025 and 2035

**S3 Table:** Additional lives saved among <1 month, 1-59 months and mothers between 2025 and 2035

*S1 Table: Additional neonatal lives saved by cause between 2025 and 2035*

| **Causes** | **2025** | **2026** | **2027** | **2028** | **2029** | **2030** | **2031** | **2032** | **2033** | **2034** | **2035** |
| --- | --- | --- | --- | --- | --- | --- | --- | --- | --- | --- | --- |
| Neonatal - Asphyxia | 0 | 49 | 96 | 140 | 182 | 223 | 265 | 306 | 347 | 388 | 427 |
| Neonatal - Prematurity | 0 | 45 | 88 | 128 | 167 | 203 | 240 | 277 | 313 | 347 | 381 |
| Neonatal - Sepsis | 0 | 33 | 63 | 90 | 116 | 139 | 162 | 184 | 204 | 224 | 242 |
| Neonatal - Pneumonia | 0 | 28 | 54 | 78 | 101 | 122 | 144 | 165 | 186 | 206 | 225 |
| Neonatal - Diarrhoea | 0 | 13 | 25 | 36 | 45 | 54 | 63 | 71 | 78 | 85 | 91 |
| Neonatal - Congenital  anomalies | 0 | 2 | 5 | 7 | 9 | 11 | 13 | 16 | 18 | 20 | 22 |
| Neonatal - Tetanus | 0 | 2 | 3 | 4 | 5 | 6 | 7 | 8 | 9 | 10 | 11 |
| Neonatal - Other | 0 | 0 | 0 | 0 | 0 | 0 | 0 | 0 | 0 | 0 | 0 |

*S2 Table: Neonatal mortality rate by year*

| **Year** | **NMR per 1,000 live births** |
| --- | --- |
| 2025 | 17 |
| 2026 | 16.68 |
| 2027 | 16.36 |
| 2028 | 16.05 |
| 2029 | 15.75 |
| 2030 | 15.46 |
| 2031 | 15.17 |
| 2032 | 14.88 |
| 2033 | 14.6 |
| 2034 | 14.33 |
| 2035 | 14.06 |

*S3 Table: Additional lives saved among <1 month, 1-59 months and mothers between 2025 and 2035*

| **Year** | **<1 month** | **1-59 months** | **Maternal lives Saved** |
| --- | --- | --- | --- |
| 2025 | 0 | 1 | 0 |
| 2026 | 172 | 133 | 8 |
| 2027 | 333 | 255 | 15 |
| 2028 | 483 | 369 | 22 |
| 2029 | 625 | 475 | 28 |
| 2030 | 759 | 573 | 34 |
| 2031 | 894 | 665 | 41 |
| 2032 | 1,027 | 754 | 48 |
| 2033 | 1,155 | 839 | 54 |
| 2034 | 1,279 | 921 | 61 |
| 2035 | 1,399 | 992 | 47 |
| **Total** | **8,126** | **5,977** | **358** |
