# Supplementary material for: Where to invest in neonatal survival programs in Nepal? A modelling study using Lives Saved Tool through scaling key interventions
Source: PLoS One. 2025 Dec 4;20(12):e0337331. doi: 10.1371/journal.pone.0337331 (PMC12677573; doi:10.1371/journal.pone.0337331)
Supplement: Attachment — Submitted filename: Response to Reviewers_R2.docx [file pone.0337331.s005.docx]

Point by point response to reviewer comments

Submission ID: [PONE-D-25-15453R1] - [EMID:316578d87e75072a]

**Where to Invest in Neonatal Survival Programs in Nepal?  A Modelling Study Using Lives Saved Tool Through Scaling Key Interventions**

Dear Editor and Reviewer,

We sincerely appreciate the time and effort you have dedicated to reviewing our manuscript. We are grateful for your insightful comments and constructive suggestions, which have significantly improved the quality of our work.

In response to the reviewers’ feedback, we have carefully addressed each point raised. Below, we outline our revisions in two parts:

1. **Reviewers’ comments** (in black font), and
2. **Authors’ responses** (in black italic font).

Additionally, we have attached a comprehensive response letter that outlines how we have incorporated the reviewers’ suggestions into the revised manuscript.

We have addressed the comments as follows:

1. ‘Response letter (Black colour bold font), and

2. ‘Authors’ RESPONSE (black colour font & italic).

All changes made to the manuscript have been highlighted in the "Revised Manuscript with Track Changes" document where insertion is in *blue italic* fonts and deletion are in ~~red strikethrough~~ fonts. A clean version of the revised manuscript has also been provided for your convenience.

We look forward to your kind consideration.

Sincerely,

Geha Nath Khanal

On behalf of co-authors

**REVIEWER 1**

**This is an important and timely paper that can contribute to reducing neonatal deaths in Nepal and monitoring progress toward the SDGs. Thank you for addressing all comments carefully. A thorough review of the language, in-text citations, and references would further strengthen the manuscript.**

*Response: We would like to thank you for your assessment and review of previous version of our manuscript. We are pleased that the previous revisions that we had made after your valuable review and feedback were satisfactory.*

*We have tried our best in reviewing the language and references as suggested and have performed a through proofreading of the manuscript to correct grammatical errors. We have checked and added some references (increased from 43 to 46 now) which were missing in the previous version of manuscript. Furthermore, we have also added supplementary table 4 which was missing in the earlier version.*

**REVIEWER 3**

**This paper provides a clear, data-driven analysis of neonatal mortality in Nepal using the LiST model to estimate the impact of scaling up national priority interventions. It is strong in its policy relevance, methodological transparency, and integration of national and global health frameworks. The analysis effectively identifies high-impact interventions and links them to actionable policy strategies. There are a few targeted areas for improvement, such as tightening the writing, polishing grammar and some phrasing, and strengthening the connection between results and policy implications, that would enhance coherence and impact.**

*Response: Thank you for your evaluation and feedback. We have tried our best to improve in grammar, sentence structure and coherence between results and policy implications as suggested.*

**Abstract**

**The abstract effectively addresses a critical public health issue - neonatal mortality in Nepal - within a clear global and national policy context. It presents well-organized sections with quantitative, model-based estimates that make the projected impact tangible and actionable. The identification of key priority interventions adds strong practical relevance and supports evidence-based decision-making.**

*Response: Thank you for your observation and evaluation.*

**There are a few minors areas of improvement you could consider:**

1. **Minor grammatical and phrasing issues need correction for clarity.**

*Response: Thank you for suggestion. We have revised it accordingly.*

2. **Methods section could briefly clarify use of proxy estimates and modelling assumptions.**

*Response: Thank you for your suggestions. We have CORRECTED accordingly.*

3. **Integrating a few points of how findings can inform policy or program implementation would strengthen conclusion.**

*Response: Thank you for suggesting this important point which was missing in the previous version of manuscript. We have CORRECTED accordingly.*

4. **It would be helpful to address the gap between projected NMR (14/1,000) and the NENAP target (11/1,000).**

*Response: Thank you for your critical observation. We have CORRECTED accordingly.*

**Introduction**

**The introduction provides a clear, data-driven rationale for the study, effectively situating neonatal mortality within both the global and national (Nepalese) context. It demonstrates strong use of recent data and policy references, highlighting Nepal’s past progress, remaining challenges, and alignment with national and international frameworks such as NENAP, SDGs, and WHO recommendations. The logical flow from problem statement to research gap and study aim is coherent and well-grounded in evidence. A few minor items that would strengthen this section:**

*Response: Thank you for your observation.*

5. **Correct minor grammatical and phrasing errors (e.g., line 111: “….effect of selected maternal and neonatal health interventions to the coverage targets set by NENAP for 2035” would be better phrased as “….effect of selected maternal and neonatal health interventions at the coverage targets set by NENAP for 2035”; e.g., line 117 “setting the evidence-based interventions in newborn survival policies and programs” is a bit awkward – perhaps use “informing” or “guiding” the integration of evidence-based interventions.**

*Response: Thank you for your observation. We have CORRECTED accordingly.*

6. **Consider streamlining a few sections to improve readability and maintain focus on key points (e.g., summarize or group Nepal’s MNH programs in lines 73-79 instead of a full list; lines 85-98 could be streamlined a bit as it repeats similar ideas about the need for targeted interventions).**

*Response: Thank you for your feedback. We have CORRECTED accordingly. (Page 4 )*

7. **Strengthen the transition to the study aim by clarifying the research gap.**

*Response: Thank you for your critical observation. We have CORRECTED accordingly. The expanded section reads as following: (Page 4, Line 106 to 111)*

“While the efficacy of these interventions individually is well documented, a critical evidence gap remains regarding the combined impact of simultaneously scaling up the entire package of NENAP priority interventions. Our study aims to provide policymakers with the evidence by quantifying how many lives could be saved and to what extent such a scale-up could close the gap between current trends and national targets.”

8. **Briefly note limitations of past interventions to justify the study’s relevance.**

*Response: Thank you for your feedback. We have CORRECTED accordingly.*

**Methods**

**The methods section clearly describes the modeling tool (LiST) and software used, providing sufficient detail about the data sources and modules involved. It explains the modeling strategy and assumptions well, including how intervention effectiveness and coverage translate into impact. The inclusion of baseline and target coverage data offers transparency and supports reproducibility. A few minor areas to improve clarity and readability:**

*Response: Thank you for your observation.*

9. **Some sentences are long and complex; breaking them into shorter, clearer statements would improve readability (e.g., lines 121–124).**

*Response: Thank you for your feedback. We have CORRECTED accordingly.*

10. **Minor grammatical issues (e.g., line 125 “affect in health outcomes” to “affect health outcomes”).**

*Response: Thank you for your feedback. We have CORRECTED accordingly.*

11. **The explanation of effectiveness and impact calculations could be streamlined to avoid redundancy (lines 159–170).**

*Response: Thank you for your feedback. We have revised accordingly. The revised section reads as follows: (Page 8, Line 161 to 172).*

The formula to calculate the impact is:

Impact = (Change in intervention coverage) X (Effectiveness of intervention) X (Affected fraction)

Effectiveness is defined as the as the proportional reduction in cause-specific mortality achievable at full coverage, was sourced from global meta-analyses and systematic reviews to ensure standardized estimates [13]. For example, zinc treatment for diarrhea has the effectiveness of 23%, which means it can prevent 23% of diarrhoeal deaths among children aged 1-59 months when implemented at 100% coverage, assuming other factors remain constant. The model assumes a proportional relationship: 50% coverage level would therefore achieve 50% of the maximum potential impact (i.e., an 11.5% reduction in diarrheal deaths in this example) [10].

12. **A short add on to clarify the rationale for selecting the 35 interventions—why these and not others? – would be helpful. For example, “We selected…….model, prioritizing those identified as key drivers of neonatal survival and aligned with national targets for 2035“ or perhaps “…focusing on those with established effectiveness and significant potential impact on neonatal, maternal, and child mortality.”**

*Response: Thank you for your feedback. We have revised accordingly. The revised section reads as follows: (Page 8, Line 174 to 176).*

We selected 35 interventions specified in the NENAP (Table 1) for inclusion in our model as these are the core monitoring indicators prioritized by the national newborn health strategy to achieve its 2035 mortality reduction targets [8].

**Results
The results section clearly quantifies the projected impact of scaling up interventions, providing specific numbers of lives saved and highlighting the most impactful interventions. It effectively uses percentages to contextualize the relative contributions of key interventions and causes of death. There is a comprehensive presentation of data. A few minor items to strengthen the results presentation:**

*Response: Thank you for your observation.*

13. **Correct the broken reference ("Error! Reference source not found") to ensure all citations and figures are properly linked and accessible (line 198).**

*Response: Thank you for your critical observation. We have CORRECTED it accordingly.*

14. **Avoid repetition in listing the four key interventions twice in consecutive sentences (lines 192–197). For example, the second reference (starting at line 194) could combine into one clear sentence: “These four interventions together could prevent more than two-thirds (70.4%) of neonatal deaths.”**

*Response: Thank you for your observation. This has been CORRECTED in the revised manuscript.*

15. **A few points of awkward phrasing: e.g., line 202 “The finding shows that birth asphyxia accounts the largest share (29.8%)” could be “Birth asphyxia accounts for the largest share (29.8%) of additional neonatal lives saved.”**

*Response: Thank you for your important observation. We have CORRECTED it accordingly.*

16. **To enhance reader understanding, include brief interpretations or implications of the results, rather than just presenting numbers.**

*Response: Thank you for your important observation. We have revised it accordingly. (Page 10-11 (Result section)).*

**Discussion**

**The discussion is comprehensive and demonstrates a strong command of both the national policy landscape and the study’s implications. It effectively links the model results to real-world challenges in Nepal’s neonatal health system, referencing existing programs, infrastructure, and health system constraints. The integration of evidence and policy history adds depth, and the section maintains alignment with the study’s objectives and global standards.**

*Response: Thank you for your evaluation.*

**A few items could be addressed to strengthen the discussion:**

17. **Consider places to streamline the discussion section and address minor grammatical issues. For example, can condense overlapping explanations (e.g., repeated statements about Nepal’s progress and NMR stagnation in lines 215–220 and 261–266) or simplify long sentences for readability.**

*Response: Thank you for your critical observation. We have revised it accordingly.*

18. **Focus the policy table (Table 3) by summarizing key milestones or grouping related initiatives rather than listing every program in detail. For instance, combine overlapping entries like CB-NCP, CB-IMCI, and CB-IMNCI.**

*Response: We sincerely appreciate this critical suggestion to streamline Table 3. We agree that focusing the table is crucial for clarity.*

*In our initial design, we considered grouping related initiatives. However, the discussion in our team, we made a deliberate choice to list key programs like CB-IMCI, CB-IMNCI, and CB-NCP separately to accurately represent the chronological evolution and specific focus of Nepal's neonatal health policy over the past two decades. Presenting them individually allows readers to see how the national strategy has shifted and refined its approach, moving from a broader child health focus (CB-IMCI) to a more newborn-specific one (CB-NCP) and integrating them to CBIMNCI later. We believe this detailed view is valuable for understanding the historical context of policy priorities. In the revised manuscript we have explained the historical development of community-based interventions and their evolution in paragraphs. The expanded section reads as following: (Page 13, Line 261-269)*

“……This achievement was partly due to community-based interventions tailored to Nepal’s unique health system of Nepal. neonatal health policies evolved from the broader child health focus of the Community-Based Integrated Management of Childhood Illness (CB-IMCI) to the more newborn-specific Community-Based Newborn Care Program (CB-NCP). These were later integrated into Community-Based Integrated Management of Neonatal and Childhood Illness (CB-IMNCI), which streamlined overlapping components to create a single, comprehensive program for managing illnesses in newborns and children under five. …..”

19. **You could strengthen the analytical discussion by more clearly distinguishing between what the LiST model adds and what prior research or policy already shows.**

*Response: Thank you for your critical feedback. We have revised it accordingly.*

**Conclusion
The conclusion effectively summarizes key findings and policy implications, maintaining consistency with the results and discussion. It ends with a clear call for a comprehensive, system-wide response, which is a strong way to close the paper. You could consider ending with a lightly stronger emphasis, such as: “Ultimately, achieving the NENAP targets will depend on integrating high-impact neonatal interventions within a strengthened, equitable health system.”**

*Response: Thank you for your valuable feedback. We have CORRECTED as suggested.*
