# Supplementary material for: Where to invest in neonatal survival programs in Nepal? A modelling study using Lives Saved Tool through scaling key interventions
Source: PLoS One. 2025 Dec 4;20(12):e0337331. doi: 10.1371/journal.pone.0337331 (PMC12677573; doi:10.1371/journal.pone.0337331)
Supplement: Attachment — Submitted filename: 14.09.2025_ Reviewer Comment Report.docx [file pone.0337331.s004.docx]

Point by point response to reviewer comments

Submission ID: [PONE-D-25-15453] - [EMID:316578d87e75072a]

**Where to Invest in Neonatal Survival Programs for Achieving the Targets of Sustainable Development Goals in Nepal?  A Modelling Study Using Lives Saved Tool Through Scaling Key Interventions**

Dear Editor and Reviewer,

We sincerely appreciate the time and effort you have dedicated to reviewing our manuscript. We are grateful for your insightful comments and constructive suggestions, which have significantly improved the quality of our work.

In response to the reviewers’ feedback, we have carefully addressed each point raised. Below, we outline our revisions in two parts:

1. **Reviewers’ comments** (in black font), and
2. **Authors’ responses** (in black italic font).

Additionally, we have attached a comprehensive response letter that outlines how we have incorporated the reviewers’ suggestions into the revised manuscript.

We have addressed the comments as follows:

1. ‘Response letter (Black colour bold font), and

2. ‘Authors’ RESPONSE (black colour font & italic).

All changes made to the manuscript have been highlighted in the "Revised Manuscript with Track Changes" document where insertion is in *blue italic* fonts and deletion are in ~~red strikethrough~~ fonts. A clean version of the revised manuscript has also been provided for your convenience.

We look forward to your kind consideration.

Sincerely,

On behalf of co-authors

**REVIEWER 1**

**The topic addressed in this paper is highly relevant for Nepal and other low- and middle-income countries (LMICs).**

*Response: Thank you for your evaluation.*

1. **Page 1 – Introduction:
   While the introduction provides useful background, it omits key factors contributing to Nepal’s progress in maternal and neonatal health. Specifically, the role of integrated programs involving family planning, water, sanitation, and hygiene (WASH), as well as community-based approaches is missing.**

*Response: Thank you for suggesting this important point which was missing in the previous version of manuscript. We have CORRECTED accordingly.*

2. **Methods Section:
   The rationale for selecting 90% coverage for all nine interventions is not clearly explained. Why were this specific threshold chosen, and why was it uniformly applied across all interventions?**

*Response: Thank you for your evaluation.*

*We have projected the targets of NENAP to be achieved by 2035. Accordingly, we have expanded the number of interventions from 9 to 35. Baseline values were extracted from recent national surveys such as DHS and NMICS. For interventions without reported baseline values in these surveys, coverage data from HMIS reports were used. Additionally, findings from regression models suggested by previous studies were applied to calculate proxy values for Nepal. Some assumptions recommended by the Spectrum manual were also incorporated for certain indicators. Detailed information on baseline and endline values, data sources for baselines, proxy indicators, and regression equations used to calculate proxy values are provided in the supplementary information.*

**The decision to assign equal weight to all nine interventions requires justification. Not all interventions may have an equivalent impact on neonatal mortality, so the use of equal weightage should be discussed.**

*Response: We sincerely appreciate this observation. To clarify, our analysis did not assume equal weighting across the nine interventions. While we had not explicitly stated this in previous manuscript, we have now clarified the methodology in the revised version.*

*Specifically:*

i. *Impact calculation follows the formula:
   Impact = (Change in intervention coverage) × (Effectiveness of intervention) × (Affected fraction)*
   - *Change in coverage is derived from baseline/endline inputs (Table 1 in our case).*
   - *Effectiveness and Affected fraction (i.e., intervention weights) are automatically determined by LiST, based on the latest evidence from peer-reviewed literature*
ii. *We have added this clarification in the Methods section to enhance clarity*

*We thank you for prompting this important clarification.*

3. **The inclusion of the Family Planning (FamPlan) and AIDS Impact Module (AIM) is unclear. Why were these modules used in the context of neonatal health and how they contribute to the analysis?**

*Response: Thank you for highlighting this important point. REVISED as highlighted. The expanded section reads as following:* (Page 6-7)

“ We conducted the modelling using Spectrum software, version 6.1 incorporating two key modules: demographic module (DemPro) and family planning module (FamPlan). The DemPro module was employed to integrate demographic projections of births and deaths, providing the population structure and fertility assumptions necessary for accurate mortality estimates. The FamPlan module estimated the impact of family planning services on birth rates and fertility patterns, which influence the number of births and subsequent mortality risks. The LiST has been previously used in Nepal to estimate the maternal, newborn and under-five childhood deaths, as well as stillbirths demonstrating its applicability in this context. ” (Line 129-138)

4. **The assumption of a 0% baseline for fetal growth restriction management and Kangaroo Mother Care (KMC) is unrealistic and should be revisited or supported with evidence.**

*Response: Thank you for your suggestions. We have revised the manuscript by updating the targets to reflect the best achievable level and selecting the 35 interventions specified in NENAP. The baseline values were derived from the nearest available data from previous national surveys or regression models, with full details on their sources and calculations provided in the supplementary information.* (Page 1-6 of Supplementary File)

5. **Key Issue Regarding SDG Target:
   Even under the assumption of achieving 90% coverage for all interventions, the SDG) target of reducing neonatal mortality rate (NMR) to 12 deaths per 1,000 live births by 2030 is not met. The manuscript does not adequately address why 90% coverage was chosen if it does not align with achieving the SDG target. Given that the title suggests alignment with SDG goals, this discrepancy should be discussed more thoroughly.**

*Response: We sincerely appreciate this critical suggestion. Incorporing the similar suggestions by Reviewer 2 as well, we have revised the baseline and targets accordingly and have been explained earlier.*

6. **Discussion Section:
   The discussion is overly lengthy and lacks focus, which may reduce reader engagement. Summarizing or condensing context-specific information to improve clarity and maintain relevance for an international audience will strengthen the paper.**

*Response: Thank you for your observation and suggestions. In the revised manuscript, we have revised the whole manuscript as suggested. All corrections have been incorporated in the revised version with tracked changed file.*

7. **Minor Revisions:**
   a. **Page 8, Lines 152–153: Citation is missing; please provide appropriate references.**

*Response: Thank you for your critical observation. We have provided appropriate reference as suggested.*

b. **Page 17: Expand “FCHV” upon first use to ensure clarity for all readers.**

*Response: Thank you for your critical observation. We have CORRECTED as suggested.*

c. **Language and Grammar:
   The manuscript would benefit from thorough language and grammar editing.**

*Response: We appreciate your valuable suggestion. The manuscript has now undergone thorough language and grammar editing to ensure grammatical accuracy and improve clarity. All corrections have been incorporated in the revised version with tracked changed file.*

**REVIEWER 2**

1. **Thank you for reaching out to review this important manuscript. The study is very relevant for Nepal considering stagnation of neonatal mortality since 2016, and need to achieve ambitious targets of Sustainable Development Goal and Nepal Every Newborn Action Plan. It would be very valuable to identify effective, feasible and affordable interventions to reduce neonatal mortality in Nepal. Use of LiST is a very good approach as it provides robust model for estimating potential lives saved due to various interventions with varied level of coverage.**

*Response: Thank you for your evaluation.*

2. **As this study is based on a mathematical modelling, it is very important for the authors to check assumptions and apply their judgement of the applicability and limitations of the model, as well as to make attempts to improve the model, values and assumptions. Though the authors have noted in the limitation section, the biggest weakness of this study is to rely on default values for 2024. As per my understanding, the default values provided by the LiST software is only for the reference purpose and the authors are encouraged to identify reliable sources of data to best estimate the value for the baseline and to use those values for the model. For example, as also noted by the authors, 0% fetal growth restriction detection and management and 0% case management of premature babies with KMC in 2024 is not reflective of the true situation. Another example is, 75.6% baseline coverage of neonatal resuscitation is over estimation of the coverage considering Nepal's institutional delivery rate, availability of skilled birth attendants during birth, skill retention of birth attendants, availability of newborn resuscitation kits in health facilities.**

*Response: We sincerely appreciate this critical observation. In the revised manuscript, we have addressed this concern by:*

i. *Revising the baseline values based on the nearest available reliable data, and*
ii. *Re-evaluating the target values in alignment with the latest policy documents*

*These adjustments have been incorporated in Table 1 to ensure greater accuracy and relevance. This has been explained earlier in the response to the concerns raised by reviewer 1.*

3. **Authors noted in the Limitation section (line 358) that "LiST relies on default effectiveness estimates derived from global or regional data" but the Spectrum Manual Page 7 guides "Make any modifications necessary to the default baseline data", which reflects that change of default value in the software is possible. As a scientific work, I strongly advise the authors to use reliable estimate for the baseline value, which otherwise will provide under/over estimation of the effect and may misguide the policy-makers and program-planners. For example, this may likely to affect the three priority interventions (case management of premature babies, management of neonatal sepsis, and neonatal resuscitation) or their order.**

*Response: We sincerely appreciate this critical observation. We have REVISED as suggested.*

4. **Also, as noted by the authors, "the assumption of uniformly scaling up all interventions 90% coverage by 2030 oversimplifies implementation challenges and may not be realistic". This is also very important aspect of the study to re-consider. I would suggest considering the targets in key policy documents such as SDG, Nepal Health Sector Strategic Plan, Nepal Every Newborn Action Plan to estimate the best possible level (target) by 2030.**

*Response: Thank you for your suggestions. In the revised manuscript, we have revised the targets to estimate the best possible level. These adjustments have been incorporated in Table 1.*

5. **The "Discussion" section is lengthy and not very coherent. Please revise it with a proper logical flow. Considering Nepal's vulnerability (e.g 2015 earthquake, COVID pandemic), please also talk about vulnerability to achieve 2030 targets and actions needed to make the newborn health system more resilient. Try to evaluate how prepared Nepal's health system is to address newborn health priorities in case of any shocks.**

*Response: Thank you for your observation and suggestions. In the revised manuscript, we have revised the whole manuscript as suggested.*

6. **As addressing these comments may require substantial changes in the manuscript, I would recommend "major revision" for this manuscript.**

*Response: Thank you for your feedback. We have tried our best to address the feedback and suggestions accordingly.*

7. **Few minor corrections:**
   a. **Line # 69-70: I would not consider Neonatal Strategy as an intervention. Its a strategic document guiding priority interventions. As Nepal still relies on 2004 Strategy for Neonatal health (though there are newer documents guiding newborn health such as Safe Motherhood and Newborn Health Roadmap and Nepal Every Newborn Action Plan). So, please differentiate between policy documents and actual interventions. Please provide an overview of existing key policy documents (with key dates of endorsement) guiding newborn health in Nepal.**

*Response: Thank you for your evaluation. CORRECTED. We have now addressed this comment in the table that has presented the policy/programs.*

b. **Line # 91: rephrase the word "advocates" as it may not fit well with WHO's mandate/role**

*Response: Thank you for your observation. CORRECTED with the word recommends instead of advocates as per suggestion.*

c. **Sentences after line# 296 is repetition of sentences from line # 267, which might be a copy-paste error. Please check and update.**

*Response: Thank you for your observation. CORRECTED*
